# 单中心109例慢性粒-单核细胞白血病患者的分子学特征分析

**DOI:** 10.3760/cma.j.issn.0253-2727.2023.05.004

**Published:** 2023-05

**Authors:** 士强 曲, 丽娟 潘, 铁军 秦, 泽锋 徐, 冰 李, 慧君 王, 琦 孙, 玉娇 贾, 承文 李, 文宇 蔡, 清妍 高, 蒙 焦, 志坚 肖

**Affiliations:** 1 中国医学科学院血液病医院（中国医学科学院血液学研究所），实验血液学国家重点实验室，国家血液系统疾病临床医学研究中心，细胞生态海河实验室，天津 300020 State Key Laboratory of Experimental Hematology, National Clinical Research Center for Blood Diseases, Haihe Laboratory of Cell Ecosystem, Institute of Hematology & Blood Diseases Hospital, Chinese Academy of Medical Sciences & Peking Union Medical College, Tianjin 300020, China; 2 天津医学健康研究院，天津 301600 Tianjin Institutes of Health Science, Tianjin 301600, China

**Keywords:** 慢性粒-单核细胞白血病, 靶向测序, 基因突变, Chronic myelomonocytic leukemia, Targeted sequencing, Gene mutation

## Abstract

**目的:**

探索慢性粒-单核细胞白血病（CMML）的基因突变特征。

**方法:**

按照WHO 2022分类，对2016年3月至2021年10月113例CMML和840例骨髓增生异常综合征（MDS）患者进行CMML重新诊断，并分析符合WHO 2022标准CMML患者的临床和分子学特征。

**结果:**

113例WHO 2016标准诊断的CMML患者有23例（20.4％）重新诊断为急性髓系白血病（AML），包括18例AML伴NPM1突变，3例AML伴KMT2A重排和2例AML伴MECOM重排。另90例患者符合WHO 2022 CMML诊断标准。840例MDS患者中有19例（2.3％）符合WHO 2022 CMML诊断标准。99％的CMML患者检出至少1种基因突变，中位突变个数为4（2，5）个。突变检出率≥10％的基因依次为：ASXL1（48％）、NRAS（34％）、RUNX1（33％）、TET2（28％）、U2AF1（23％）、SRSF2（21.1％）、SETBP1（20％）、KRAS（17％）、CBL（16％）和DNMT3A（11％）。配对分析显示SRSF2同ASXL1（*OR*＝4.129，95％ *CI* 1.481～11.510，*Q*＝0.007）和TET2（*OR*＝5.276，95％ *CI* 1.979～14.065，*Q*＝0.001）常为共存突变。SRSF2和TET2常出现于老年（≥60岁）增殖型CMML（MP-CMML）患者。U2AF1同TET2（*OR*＝0.174，95％ *CI* 0.038～0.791，*Q*＝0.024）常成互斥关系，易见于年轻（<60岁）发育异常型CMML（MD-CMML）患者。单核细胞绝对值计数（AMoC）≥1×10^9^/L和<1×10^9^/L两组患者比较比较，前者有更高的中位发病年龄（60岁对47岁，*P*<0.001）、WBC（15.9×10^9^/L对4.4×10^9^/L，*P*<0.001）、单核细胞比例（21.5％对15％，*P*＝0.001）和HGB水平（86 g/L对74 g/L，*P*＝0.014）。TET2突变（*P*＝0.021）和SRSF2突变（*P*＝0.011）更常见于AMoC≥1×10^9^/L组，而U2AF1突变（*P*<0.001）更常见于AMoC<1×10^9^/L组。两组间的其他基因突变频率差异无统计学意义。

**结论:**

按照WHO 2022分类，约20％的CMML病例诊断时的AMoC<1×10^9^/L，MD-CMML和MP-CMML有不同的分子学特征。

## Abstract (en)

**Objective:**

To explore the molecular features of chronic myelomonocytic leukemia（CMML）.

**Methods:**

According to 2022 World Health Organization（WHO 2022）classification, 113 CMML patients and 840 myelodysplastic syndrome（MDS）patients from March 2016 to October 2021 were reclassified, and the clinical and molecular features of CMML patients were analyzed.

**Results:**

Among 113 CMML patients, 23（20.4％）were re-diagnosed as acute myeloid leukemia（AML）, including 18 AML with NPM1 mutation, 3 AML with KMT2A rearrangement, and 2 AML with MECOM rearrangement. The remaining 90 patients met the WHO 2022 CMML criteria. In addition, 19 of 840（2.3％）MDS patients met the WHO 2022 CMML criteria. At least one gene mutation was detected in 99％ of CMML patients, and the median number of mutations was 4. The genes with mutation frequency ≥10％ were: ASXL1（48％）, NRAS（34％）, RUNX1（33％）, TET2（28％）, U2AF1（23％）, SRSF2（21.1％）, SETBP1（20％）, KRAS（17％）, CBL（15.6％）and DNMT3A（11％）. Paired analysis showed that SRSF2 was frequently co-mutated with ASXL1（*OR*＝4.129, 95％ *CI* 1.481–11.510, *Q*＝0.007）and TET2（*OR*＝5.276, 95％ *CI* 1.979–14.065, *Q*＝0.001）. SRSF2 and TET2 frequently occurred in elderly（≥60 years）patients with myeloproliferative CMML（MP-CMML）. U2AF1 mutations were often mutually exclusive with TET2（*OR*＝0.174, 95％ *CI* 0.038–0.791, *Q*＝0.024）, and were common in younger（<60 years）patients with myelodysplastic CMML（MD-CMML）. Compared with patients with absolute monocyte count（AMoC）≥1×10^9^/L and <1×10^9^/L, the former had a higher median age of onset（60 years old *vs* 47 years old, *P*<0.001）, white blood cell count（15.9×10^9^/L *vs* 4.4×10^9^/L, *P*<0.001）, proportion of monocytes（21.5％ *vs* 15％, *P*＝0.001）, and hemoglobin level（86 g/L *vs* 74 g/L, *P*＝0.014）. TET2 mutations（*P*＝0.021）and SRSF2 mutations（*P*＝0.011）were more common in patients with AMoC≥1×10^9^/L, whereas U2AF1 mutations（*P*<0.001）were more common in patients with AMoC<1×10^9^/L. There was no significant difference in the frequency of other gene mutations between the two groups.

**Conclusion:**

According to WHO 2022 classification, nearly 20％ of CMML patients had AMoC<1×10^9^/L at the time of diagnosis, and MD-CMML and MP-CMML had different molecular features.

慢性粒-单核细胞白血病（CMML）是以克隆性单核细胞增多为特征表现的一类髓系肿瘤[1]。以往研究显示80％以上的CMML患者存在至少1种TET2、SRSF2或ASXL1突变，其中TET2双等位基因突变和TET2-SRSF2共存突变被视为CMML相对特异的分子标志[2]。2016年第4版WHO CMML诊断标准要求外周血单核细胞绝对计数（AMoC）≥1×10^9^/L以及单核细胞比例≥10％[3]。2022年第5版WHO诊断标准将CMML的AMoC诊断界值降至≥0.5×10^9^/L，将近年新提出的寡单核细胞CMML（OM-CMML）纳入CMML的诊断范畴[4]。我们按照WHO 2022分类对近年来收治的符合WHO 2016分类标准的CMML患者进行重新诊断，并分析CMML患者的临床和分子学特征。

## 病例与方法

1. 病例：本研究纳入自2016年3月至2021年10月在我中心按照WHO 2016分类标准[3]诊断的953例初诊患者，其中包含113例CMML和840例骨髓增生异常综合征（MDS）。按照WHO 2022分类[4]重新进行CMML诊断，所有患者均进行了骨髓+外周血涂片分类和骨髓活检+嗜银染色。

2. 外周血单核细胞比例界定：外周血单核细胞比例以血涂片人工分类为准。血涂片经瑞氏染色，计数200个白细胞。幼稚单核细胞作为原始细胞等同细胞。单核细胞包含正常（成熟）和异常（不成熟）单核细胞[5]–[6]。

3. 染色体核型分析：骨髓细胞经过24 h短期培养，收集细胞常规制片，R显带后进行核型分析。核型描述符合人类细胞遗传学国际命名体制（ISCN2013）[7]。克隆性核型异常判断标准：同一染色体缺失出现在至少3个中期分裂相，同一染色体获得或结构异常出现在至少2个中期分裂相。复杂异常定义为同一标本存在3种及以上克隆性异常。对少于10个分裂相的正常核型不做评估。

4. 靶向测序：从骨髓单个核细胞提取基因组DNA，针对114个血液肿瘤相关基因编码序列进行靶向深度测序，使用Illumina标准方案制备文库。平均基因覆盖率为98.1％。平均测序深度1 310×。具体测序方法参见我们此前文献报道[8]。男性患者X连锁基因的变异等位基因频率（VAF）值减半。

5. 预后评估：参照CMML特异性预后积分系统（CPSS）进行患者预后分层[9]。CPSS包含4项预后参数：①CPSS细胞遗传学分组：低危（单纯-Y、正常核型）（0分），中危（其他核型）（1分），高危（+8、7号染色体异常、复杂核型）（2分）；②WHO分型：CMML-2型（1分）；③FAB分型：增殖型CMML（1分）；④红细胞输注依赖：4个月内每8周至少输1次红细胞（1分）。根据积分将预后分为四组：低危组（0分）；中危1组（1分）；中危2组（2～3分）；高危组（4～5分）。

6. 统计学处理：基线数值为连续变量的以中位数（*IQR*）描述，为分类变量的以频数（百分比）描述。对于连续变量（数据不符合正态分布），通过Mann-Whitney *U*检验进行组间比较。对于分类变量，通过*χ*^2^检验或Fisher确切概率法进行比较。基因间的配对关联通过多重假设检验校正的Fisher确切检验进行评估（*Q*<0.05为差异有统计学意义）。双侧*P*<0.05被认为差异具有统计学意义。使用R 4.0.2和SPSS 22.0软件包进行统计分析。

## 结果

1. 病例诊断：113例WHO 2016标准诊断的CMML患者有23例重新诊断为急性髓系白血病（AML），包括18例AML伴NPM1突变（既往诊断包括3例CMML-0、4例CMML-1、11例CMML-2），3例AML伴KMT2A重排（既往诊断包括1例CMML-0、2例CMML-1）和2例AML伴MECOM重排（既往诊断包括1例CMML-0、1例CMML-1）。另90例患者符合WHO 2022 CMML诊断标准，既往诊断包括40例CMML-0、28例CMML-1和22例CMML-2（图1）。

**图1:**
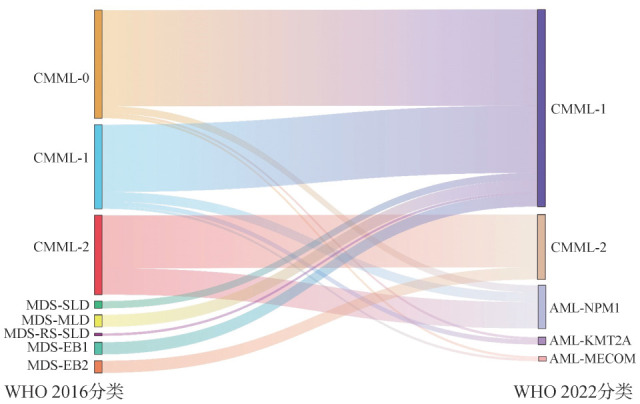
WHO 2016分类和WHO 2022分类中慢性粒-单核细胞白血病（CMML）患者亚型之间的关系 **注** MDS-SLD：骨髓增生异常综合征伴单系发育异常；MDS-RS-SLD：骨髓增生异常综合征伴环状铁粒幼红细胞和单系发育异常；MDS-MLD：骨髓增生异常综合征伴多系发育异常；MDS-EB1：骨髓增生异常综合征伴原始细胞过多1型；MDS-EB2：骨髓增生异常综合征伴原始细胞过多2型；AML-MECOM：急性髓系白血病伴MECOM基因重排；AML-KMT2A：急性髓系白血病伴KMT2A基因重排；AML-NPM1：急性髓系白血病伴NPM1基因重排

840例MDS患者中有19例符合WHO 2022 CMML诊断标准，既往诊断：3例MDS伴单系发育异常，5例MDS伴多系发育异常，1例MDS伴环状铁粒幼红细胞及单系发育异常，5例MDS伴原始细胞过多1型，5例MDS伴原始细胞过多2型（图1）。

按WHO 2022 CMML标准重新诊断的109例CMML患者，按照原始细胞计数划分为82例CMML-1和27例CMML-2。按照WBC划分为55例（50.5％）发育异常型CMML（MD-CMML）和54例（49.5％）增殖型CMML（MP-CMML）。

2. 患者基线特征：109例CMML患者的中位年龄为59（49，66）岁，男性75例（69％）。外周血中位WBC为12.6（5.3，27.5）×10^9^/L，AMoC为2.6（1.2，6.3）×10^9^/L，单核细胞比例为19％（14％，28％），HGB水平为84（65，110）g/L，PLT为59.0（31.5，118.5）×10^9^/L。101例患者有可评估的染色体核型结果，检出异常核型30例（29.7％），最常见的异常核型分别为：+8（10％）、−7（5％）、20q−（3％）和+21（2％）。CPSS危险分层：低危组12例（11％）、中危1组37例（34％）、中危2组48例（44％）、高危组12例（11％）。

对比AMoC≥1×10^9^/L和<1×10^9^/L两组患者的基线特征，前者有更高的中位发病年龄（60岁对47岁，*P*<0.001）、WBC（15.9×10^9^/L对4.4×10^9^/L，*P*<0.001）、单核细胞比例（21.5％对15％，*P*＝0.001）和HGB水平（86 g/L对74 g/L，*P*＝0.014）。两组的骨髓原始细胞比例、异常核型检出率、CPSS预后分组则差异无统计学意义（表1）。

**表1:** AMoC≥1×10^9^/L与<1×10^9^/L的符合WHO 2022慢性粒-单核细胞白血病（CMML）诊断标准患者基线特征比较

基线特征	AMoC≥1×10^9^/L组（90例）	AMoC<1×10^9^/L组（19例）	统计量	*P*值
中位年龄（岁）	60（54，67）	47（40，56）	−3.521	<0.001
男性[例（%）]	63（70.0）	12（63.2）	Fisher	0.592
中位WBC（×10^9^/L）	15.9（8.1，33.2）	4.4（3.4，4.8）	−5.847	<0.001
中位单核细胞比例（%）	21.5（15，30）	15（12，20）	−3.179	0.001
中位HGB（g/L）	86（70，112）	74（60，93）	−2.449	0.014
中位PLT（×10^9^/L）	59（32，120）	66（30，113）	−0.060	0.952
中位骨髓原始细胞
比例（%）	4.0（2.0，8.5）	6.5（2.5，9.0）	−0.580	0.562
异常核型[例（%）]	23（27.4）	7（41.2）	Fisher	0.256
突变检出率[例（%）]	89（98.9）	19（100.0）	Fisher	1.000
中位突变个数	4（3，6）	4（2，5）	−0.630	0.529
CPSS分组[例（%）]			−0.697	0.486
高危	9（10.0）	3（15.8）		
中危2	42（46.7）	6（31.6）		
中危1	31（34.4）	6（31.6）		
低危	8（8.9）	4（21.0）		

**注** AMoC：单核细胞绝对计数；CPSS：CMML特异性预后积分系统，核型不可评估的患者按照正常核型计算风险评分

3. 分子学特征：99％（108/109）的CMML患者检出至少1种基因突变，中位突变个数为4（2，5）个。突变检出率≥10％的基因依次为：ASXL1（52例，48％）、NRAS（37例，34％）、RUNX1（36例，33％）、TET2（30例，28％）、U2AF1（25例，23％）、SRSF2（23例，21％）、SETBP1（22例，20％）、KRAS（19例，17％）、CBL（17例，16％）和DNMT3A（12例，11％）（图2）。参与表观调控和RNA剪接相关基因的中位VAF值通常高于40％：EZH2为72.7％（47.4％，97％）、TET2为48.4％（46.3％，50.4％）、SRSF2为45.2％（41.4％，48.4％）、U2AF1为44.7％（40.8％，47.5％）、SF3B1为44.5％（41.3％，47.3％）、ASXL1为43.9％（39.3％，46.6％）、DNMT3A为42.6％（37.5％，47.3％）。而参与信号转导相关基因的中位VAF值通常低于40％：NRAS为34.4％（6.6％，42.1％）、KRAS为18.6％（3.5％，33.2％）、JAK2为2.9％（2.4％，37.5％）、PTPN11为2.6％（1.6％，9.9％）（图3）。

**图2:**
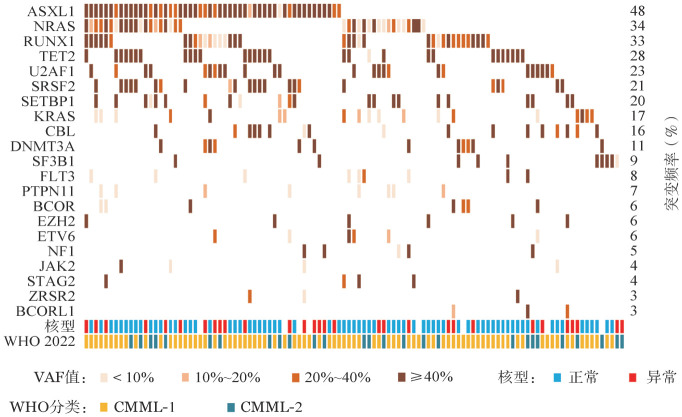
109例符合WHO 2022慢性粒-单核细胞白血病（CMML）诊断标准患者的基因突变特征

**图3:**
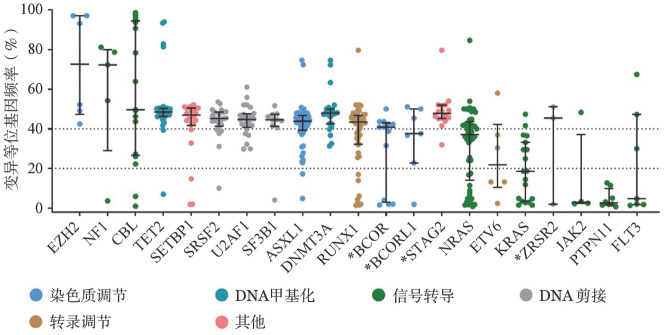
符合WHO 2022慢性粒-单核细胞白血病（CMML）诊断标准患者常见突变基因的变异等位基因频率（*代表X连锁基因）

AMoC ≥1×10^9^/L的CMML最常见的突变基因依次为：ASXL1（45例，50.0％）、NRAS（31例，34.4％）、RUNX1（31例，34.4％）、TET2（29例，32.2％）、SRSF2（23例，25.6％）、SETBP1（17例，18.9％）、CBL（16例，17.8％）、KRAS（15例，16.7％）、U2AF1（14例，15.6％）和FLT3（9例，10.0％）。AMoC<1×10^9^/L的CMML最常见的突变基因依次为：U2AF1（11例，57.9％）、ASXL1（7例，36.8％）、NRAS（6例，31.6％）、RUNX1（5例，26.3％）、SETBP1（5例，26.3％）、KRAS（4例，21.1％）、DNMT3A（4例，21.1％）、PTPN11（3例，15.8％）、SF3B1（2例，11％）、BCOR（2例，11％）和ETV6（2例，11％）。两组对比，TET2突变（*P*＝0.021）和SRSF2突变（*P*＝0.011）更常见于AMoC≥1×10^9^/L组，而U2AF1突变（*P*<0.001）更常见于AMoC<1×10^9^/L组。两组间的其他基因突变频率差异无统计学意义（图4）。

**图4:**
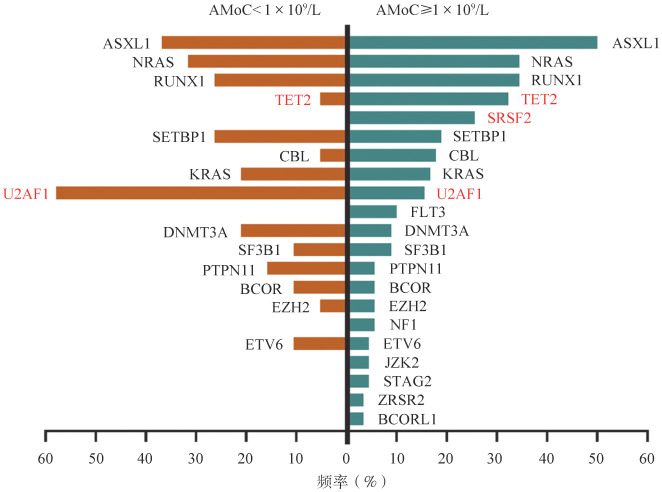
AMoC≥1×10^9^/L与AMoC<1×10^9^/L的符合WHO 2022 CMML诊断标准患者突变基因频率比较 AMoC：单核细胞绝对计数；CMML：慢性粒-单核细胞白血病

4. 基因与临床参数之间的配对分析：我们将CMML患者突变频率≥5％的基因与常见的临床预后参数进行配对分析。结果显示SRSF2同ASXL1（*OR*＝4.129，95％ *CI* 1.481～11.510，*Q*＝0.007）和TET2（*OR*＝5.276，95％ *CI* 1.979～14.065，*Q*＝0.001）常为共存突变。SRSF2和TET2常出现于老年（≥60岁）MP-CMML患者。U2AF1同TET2（*OR*＝0.174，95％ *CI* 0.038～0.791，*Q*＝0.024）常成互斥关系，而同SETBP1（*OR*＝3.072，95％ *CI* 1.121～8.420，*Q*＝0.029）常为共存突变，易见于年轻（<60岁）MD-CMML患者，同严重的贫血和8号染色体三体相关。此外，NRAS和ASXL1突变更常见于MP-CMML表型，而DNMT3A和SF3B1突变则更常见于MD-CMML表型（图5）。

**图5:**
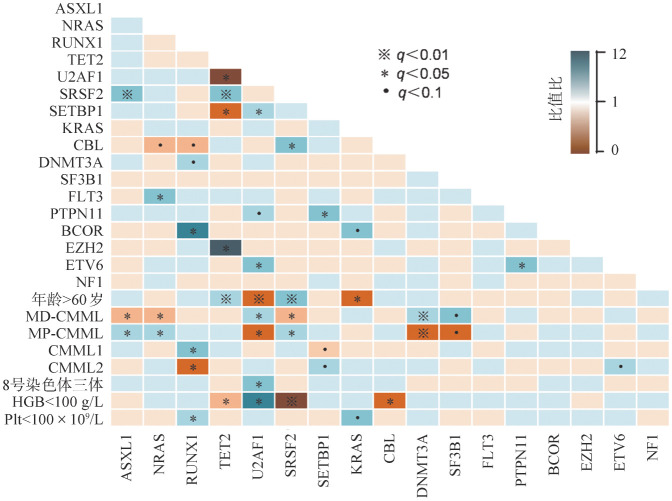
109例符合WHO 2022慢性粒-单核细胞白血病（CMML）诊断标准患者重现性突变基因与临床参数之间的配对关联

## 讨论

WHO 2022分类标准对WHO 2016版的髓系肿瘤分类进行了修改，新的分类更加强调遗传学在诊断分类中的作用。在一组由遗传学异常界定的AML中，除了BCR-ABL1融合和CEBPA突变以外，当存在其他确定的遗传学异常时，AML诊断将不再对原始细胞比例作要求[4]。这使得以往伴有这些确定遗传学异常且原始细胞比例低于20％的CMML修订诊断为AML。我们这组有23例（20％）患者重新诊断为AML，其中最常见的是伴有NPM1突变的患者。以往的一些研究中，NPM1突变的CMML往往会快速进展为AML，而接受AML样的诱导化疗方案有更高的缓解率[10]–[12]。我们这组NPM1突变患者有61％在就诊时即诊断为CMML-2，也间接反映了这组疾病快速进展的生物学特性。

2017年提出了OM-CMML的概念[13]，2019年CMML及其前驱疾病的维也纳专家共识提出了其具体诊断标准[5]。OM-CMML按照WHO 2016分类多数诊断为MDS或骨髓增生异常/骨髓增殖性肿瘤-未分类[5]。多项研究显示OM-CMML同典型CMML有相似的分子学特征和外周血单核细胞亚群分布，部分OM-CMML会最终进展为典型CMML[14]–[16]。因此，WHO 2022分类下调了CMML的AMoC诊断阈值，将OM-CMML纳入到CMML的诊断范畴中。我们对840例初诊MDS患者进行筛查，仅19例患者符合WHO 2022 CMML诊断标准，反映出AMoC<1×10^9^/L的CMML在临床中并不多见，绝大多数CMML患者就诊时AMoC≥1×10^9^/L。在临床特征方面，AMoC<1×10^9^/L的CMML患者初次就诊时更加年轻、贫血更严重，这在以往的一些研究中也有报道，但不同中心的结果并不完全一致[13]–[16]。

以往文献报道欧美CMML患者最常见的突变基因是TET2、SRSF2和ASXL1[17]–[20]，突变频率分别为60％、50％和40％左右[5]。我们按照WHO 2022分类标准诊断的这组CMML患者最常见的突变基因则分别是ASXL1、NRAS和RUNX1，可能的解释是我们这组患者CPSS较高危组（中危2+高危）患者占了55％，代表了相对晚期的一组CMML患者，而CPSS分子预后积分系统中，NRAS、RUNX1、ASXL1和SETBP1均为不良的分子预后标志，容易出现在更高危的患者中[21]。我们曾对145例符合WHO 2008 CMML诊断标准的患者进行了一代测序研究，ASXL1、TET2、SRSF2和SETBP1等4种基因的突变频率分别为45％、32％、29％和18％[22]。而本次研究显示以上4种基因的突变频率分别为48％、28％、21％和20％，TET2和SRSF2突变频率较前下降的可能原因是新的诊断标准增加了MD-CMML患者比例（50.5％对35.0％，*P*＝0.014）[22]，而配对分析显示TET2和SRSF2更多见于MP-CMML。至于我们的两项研究结果中TET2和SRSF2突变频率均低于欧美CMML患者的原因，可能的解释是不同的发病年龄决定了突变基因的分布特征。欧美CMML患者中位发病年龄通常在70～75岁[1],[5]，而我们这两项研究患者的中位发病年龄分别为59岁和63岁[22]。配对分析显示TET2和SRSF2常见于老年（≥60岁）CMML患者，而U2AF1突变多见于年轻（<60岁）CMML患者。

对比AMoC≥1×10^9^/L和<1×10^9^/L两组患者的分子学特征，最大的差异是前者的TET2和SRSF2突变率显著高于后者，而后者的U2AF1突变率显著高于前者。TET2和SRSF2共存突变是相对特异的CMML分子标志[2]。而U2AF1则是MDS常见的驱动基因。我们之前的研究显示U2AF1是中国MDS患者最常见的突变基因[8],[23]。对于AMoC<1×10^9^/L的CMML患者分子学特征与之前国外研究结果不相一致的原因，可能的解释是我们这组患者代表了伴有单核细胞进展的MDS，而非原发性的CMML。当MDS疾病进展阶段，获得了NRAS、RUNX1、ASXL1、SETBP1等CMML的高危突变，演变成CMML表型。在之前的一些文献已有相应报道，MDS或原发性骨髓纤维化进展阶段出现了单核细胞增多的临床表型[24]–[26]。

综上，按照WHO 2022诊断标准，约20％的CMML患者诊断时的AMoC<1×10^9^/L，MD-CMML和MP-CMML有不同的分子学特征。新分类对CMML患者的预后和治疗选择的影响仍有待进一步研究。

## References

[1] Patnaik MM, Tefferi A (2022). Chronic myelomonocytic leukemia: 2022 update on diagnosis, risk stratification, and management[J]. Am J Hematol.

[2] Itzykson R, Kosmider O, Renneville A (2013). Clonal architecture of chronic myelomonocytic leukemias[J]. Blood.

[3] Arber DA, Orazi A, Hasserjian R (2016). The 2016 revision to the World Health Organization classification of myeloid neoplasms and acute leukemia[J]. Blood.

[4] Khoury JD, Solary E, Abla O (2022). The 5th edition of the World Health Organization Classification of Haematolymphoid Tumours: Myeloid and Histiocytic/Dendritic Neoplasms[J]. Leukemia.

[5] Valent P, Orazi A, Savona MR (2019). Proposed diagnostic criteria for classical chronic myelomonocytic leukemia (CMML), CMML variants and pre-CMML conditions[J]. Haematologica.

[6] 中华医学会血液学分会白血病淋巴瘤学组 (2021). 慢性粒-单核细胞白血病诊断与治疗中国指南(2021年版)[J]. 中华血液学杂志.

[7] Shaffer LG, McGowan-Jordan J, Schmid M (2013). ISCN 2013: an international system for human cytogenetic nomenclature (2013) [M].

[8] Li B, Liu J, Jia Y (2018). Clinical features and biological implications of different U2AF1 mutation types in myelodysplastic syndromes[J]. Genes Chromosomes Cancer.

[9] Such E, Germing U, Malcovati L (2013). Development and validation of a prognostic scoring system for patients with chronic myelomonocytic leukemia[J]. Blood.

[10] Peng J, Zuo Z, Fu B (2016). Chronic myelomonocytic leukemia with nucleophosmin (NPM1) mutation[J]. Eur J Haematol.

[11] Vallapureddy R, Lasho TL, Hoversten K (2017). Nucleophosmin 1 (NPM1) mutations in chronic myelomonocytic leukemia and their prognostic relevance[J]. Am J Hematol.

[12] Montalban-Bravo G, Kanagal-Shamanna R, Sasaki K (2019). NPM1 mutations define a specific subgroup of MDS and MDS/MPN patients with favorable outcomes with intensive chemotherapy[J]. Blood Adv.

[13] Geyer JT, Tam W, Liu YC (2017). Oligomonocytic chronic myelomonocytic leukemia (chronic myelomonocytic leukemia without absolute monocytosis) displays a similar clinicopathologic and mutational profile to classical chronic myelomonocytic leukemia[J]. Mod Pathol.

[14] Calvo X, Garcia-Gisbert N, Parraga I (2020). Oligomonocytic and overt chronic myelomonocytic leukemia show similar clinical, genomic, and immunophenotypic features[J]. Blood Adv.

[15] Montalban-Bravo G, Kanagal-Shamanna R, Guerra V (2021). Clinical outcomes and influence of mutation clonal dominance in oligomonocytic and classical chronic myelomonocytic leukemia[J]. Am J Hematol.

[16] Valent P (2020). Oligo-monocytic CMML and other pre-CMML states: Clinical impact, prognostication and management[J]. Best Pract Res Clin Haematol.

[17] Mason CC, Khorashad JS, Tantravahi SK (2016). Age-related mutations and chronic myelomonocytic leukemia[J]. Leukemia.

[18] Patel BJ, Przychodzen B, Thota S (2017). Genomic determinants of chronic myelomonocytic leukemia[J]. Leukemia.

[19] Palomo L, Meggendorfer M, Hutter S (2020). Molecular landscape and clonal architecture of adult myelodysplastic/myeloproliferative neoplasms[J]. Blood.

[20] Coltro G, Mangaonkar AA, Lasho TL (2020). Clinical, molecular, and prognostic correlates of number, type, and functional localization of TET2 mutations in chronic myelomonocytic leukemia (CMML)-a study of 1084 patients[J]. Leukemia.

[21] Elena C, Gallì A, Such E (2016). Integrating clinical features and genetic lesions in the risk assessment of patients with chronic myelomonocytic leukemia[J]. Blood.

[22] Cui Y, Tong H, Du X (2015). Impact of TET2, SRSF2, ASXL1 and SETBP1 mutations on survival of patients with chronic myelomonocytic leukemia[J]. Exp Hematol Oncol.

[23] 李 冰, 王 静雅, 刘 晋琴 (2017). 靶向测序检测511例骨髓增生异常综合征患者基因突变[J]. 中华血液学杂志.

[24] Wang SA, Galili N, Cerny J (2006). Chronic myelomonocytic leukemia evolving from preexisting myelodysplasia shares many features with de novo disease[J]. Am J Clin Pathol.

[25] Selimoglu-Buet D, Badaoui B, Benayoun E (2017). Accumulation of classical monocytes defines a subgroup of MDS that frequently evolves into CMML[J]. Blood.

[26] Boiocchi L, Espinal-Witter R, Geyer JT (2013). Development of monocytosis in patients with primary myelofibrosis indicates an accelerated phase of the disease[J]. Mod Pathol.

